# Preliminary investigation of the transmission of tuberculosis between farmers and their cattle in smallholder farms in northwestern Ethiopia: a cross-sectional study

**DOI:** 10.1186/s13104-016-2349-z

**Published:** 2017-01-07

**Authors:** Anwar Nuru, Gezahegne Mamo, Aboma Zewude, Yitayal Mulat, Gashaw Yitayew, Aschalew Admasu, Girmay Medhin, Rembert Pieper, Gobena Ameni

**Affiliations:** 1 Aklilu Lemma Institute of Pathobiology, Addis Ababa University, P.O. Box 1176, Addis Ababa, Ethiopia; 2 Bahir Dar Regional Health Research Laboratory Centre, P.O. Box 641, Bahir Dar, Ethiopia; 3 College of Veterinary Medicine and Agriculture, Addis Ababa University, P.O. Box 34, Debre Zeit, Ethiopia; 4 College of Veterinary Medicine and Animal Sciences, University of Gondar, P.O. Box 346, Gondar, Ethiopia; 5 Felegehiwot Comprehensive Specialized Hospital, P. O. Box: 47, Bahir Dar, Ethiopia; 6 J. Craig Venter Institute, 9704 Medical Center Drive, Rockville, MD USA

**Keywords:** Cattle, Farmer, Transmission, Mycobacterium bovis, Tuberculous lymphadenitis

## Abstract

**Background:**

The feeding habits and close physical contact between Ethiopian farmers and their cattle promote the transmission of tuberculosis (TB) between the farmers and their cattle. This study aimed to investigate the transmission of TB between farmers and their cattle in smallholder farms in northwestern Ethiopia.

**Results:**

A total of 70 human TB lymphadenitis (TBLN) cases visiting the Felegehiwot Comprehensive Specialized Hospital in Bahir Dar City and 660 cattle were investigated. Half of the cattle were owned by households with TB cases, and the remaining half by TB free households. Among the 70 human TBLN patients interviewed, 65.7% (46 out of 70) of the respondents were not aware of zoonotic TB, and 67.1% (47/70) of them consumed raw milk. Positive cultures of TB were obtained in 40 of the 70 cases where TBLN tests were positive with fine needle aspiration cytology. Spoligotyping resulted in 31 different patterns, of which 25 isolates were Mycobacterium (*M.*) *tuberculosis*, and the remaining were *M. africanum* (4 isolates) and *M. bovis* (2 isolates). None of the animals showed positive test results for bovine TB by comparative intradermal tuberculin test.

**Conclusions:**

Based on the identification of *M. bovis* from two patients diagnosed with TBLN, we obtained preliminary evidence of zoonotic transmission of TB in northwestern Ethiopia. We did not identify a direct route of transmission between cattle and its owners. This is the objective of further investigations.

## Background

The *Mycobacterium tuberculosis* complex (MTBC) consists of closely related species such as *Mycobacterium* (*M.*) *tuberculosis, M. bovis*, *M. africanum, M. canettii*; *M. caprae*, *M. pinnipedii* and *M. microti* [1]. *M. bovis* primarily causes bovine tuberculosis (BTB) in animals and its transmission to humans has a public health importance [2]. A review of zoonotic TB [3] estimates the proportion of human TB cases due to *M. bovis* to account for 3.1% of all forms of TB; 2.1% of pulmonary and 9.4% of extra-pulmonary forms. Consumption of unpasteurized milk from infected cows [4] and aerosol transmission, especially where human share common premises with infected animals [5] are considered the usual mode of transmission of TB from animals to humans.

Since there are no effective animal TB control programs and lack of routine milk pasteurization procedure in low income countries [6, 7], the prevalence of human TB due to *M. bovis* is likely to be higher in countries where BTB is endemic in cattle [5] and high prevalence of human immunodeficiency virus [8]. In Ethiopia, the presence of BTB in cattle [9–18] and human due to *M. bovis* [9, 19, 20] were reported previously. After isolating *M. tuberculosis* from animals it was also suggested the presence of human to animal transmission in Ethiopia [21–23]. However, the direct link of transmission between the specific cattle and its owners were not confirmed. Identification of similar strains of MTBC species both in humans and animals using molecular techniques was therefore essential to provide evidence based suggestions on the occurrence of transmission. This study was formulated to investigate the transmission of zoonotic TB between cattle and its owners in smallholder farms in northwestern Ethiopia.

This study did not confirm a direct link of transmission of TB between specific cattle and its owners in the present study. However, there is preliminary evidence of zoonotic transmission of TB in the smallholder farms of northwestern Ethiopia. This is because molecular characterization by spoligotyping identified strains of *M. bovis* from two human TB lymphadenitis (TBLN) cases. The majority of the respondents (including the two individuals with *M. bovis*) were identified by the questionnaire as consumers of raw milk and unaware of BTB. None of the animals showed positive test results for TB by comparative intradermal tuberculin (CIDT) test.

## Methods

### Study design

The study design was cross sectional. Human patients diagnosed with TBLN at the Felegehiwot Comprehensive Specialized Hospital (located in Bahir Dar City) and their cattle traced to the village of origin of the patients were tested for symptoms of TB, microbial culture evidence of the *M. tuberculosis* complex species, and lineage using a genetic test. In parallel, a comparable number of cattle owned by TB free households, who live in the proximity of the TBLN patients, were examined. TBLN patients were human patients with enlarged lymph nodes, and who were clinically and cytologically diagnosed as TBLN. TB free households were defined as follows: absence of TB suggestive clinical signs and absence of symptoms at the time of meeting with the investigator collecting data from the consenting individuals. Clinical signs and symptoms included a history of fever and/or cough of greater than two weeks of duration, a failure to gain weight, a loss of appetite, a decline in weight, symptoms of extra-pulmonary TB (EPTB) such as swollen lymph nodes, and the absence of confirmed TB cases in any member of the household during the last 10 years.

### Sample and data collection from human subjects

Basic demographic data, and information related to awareness on BTB and its public health implication, and consumption habit of milk and meat were collected from each TBLN patient through an interview using semi-structured questionnaire. Collection of fine needle aspiration (FNA) sample and FNA cytology (FNAC) was done by the pathologist. FNAC was used for the diagnosis of mycobacterial lymphadenitis. FNAC positive samples were drained in a tube containing 1 ml phosphate buffer saline (PBS) solution and used for mycobacterial culture.

### Comparative intradermal tuberculin

CIDT was carried out to test cattle for BTB according to the OIE protocol [24]. After two sites, 12 cm apart, on skin of middle third of the neck were shaved and the thickness of each was measured with a caliper, two types of purified protein derivative (PPDs) (supplied by Prionics Lelystad B. V., The Netherlands) were injected intradermally into the two sites. One site was injected with an aliquot of 0.1 ml (ml) of 2500 IU/ml bovine-PPD (B-PPD) and the other was injected with 0.1 ml of 2500 IU/ml Avian-PPD (A-PPD). The skin thickness at each injection site was measured again after 72 h. An animal was considered to be positive for BTB if the skin reaction at the PPD-B site minus the skin reaction at the PPD-A is ≥4 mm.

### Culture and spoligotyping

TB cultures were performed using the procedure described by the National TB and Leprosy Control Programme Guideline [25] that was adopted from WHO guideline [26]. Briefly, FNA samples collected from TBLN patients in this study were processed and inoculated on duplicate Lowenstein–Jensen (LJ) slants, one supplemented with pyruvate and the other with glycerol. All the tubes were incubated at 37 °C and slants with no growth at week 8 were considered negative. Bacterial colonies from culture-positive samples were Ziehl-Neelsen stained to identify acid fast bacilli (AFB). Cultures positive for AFB were inactivated by heating at 85 °C for 45 min in a water bath and spoligotyped as previously described [20].

### Data analysis

All the statistical data were analyzed by STATA statistical software, version 12 (Stata Corp., Collage station, TX, USA). Chi square test, bivariate and multivariable logistic regression analysis were applied for selected demographic factors verses awareness, and milk and meat consumption habits. Statistical significance was assumed if the confidence interval (CI) did not include one among its values or whenever P value was less than 5%.

The generated spoligotype data were converted into binary and octal formats and entered into the open source spoligotype database available at the website http://www.pasteur-guadeloupe.fr:8081/SITVIT_ONLINE/tools.jsp to establish the lineage, sublineage, and the shared international spoligotype (SIT) number. In addition, the online tool “Run TB-Lineage” (http://tbinsight.cs.rpi.edu/run_tb_lineage.html) was used to predict the major lineages to which the strains belong by a conformal Bayesian network (CBN) analysis.

## Ethical considerations

The study was approved by Ethical Review Board (Ref. Number IRB/05-02/2013) of the Aklilu Lemma Institute of Pathobiology, Addis Ababa University. All human subjects were given written consent to participate in the study and their cattle to be part of the study.

## Results

### Level of awareness of the farmers and their food consumption habits

Among the 70 human TBLN patients interviewed, 65.7% (46/70) of the individuals did not know that cattle can transmit BTB (it stands for bovine tuberculosis caused by *M. bovis* in cattle and other mammals including man). The majority (67.1%, 47/70) of the patients had the habit of consuming raw milk. 54.3% (38/70) of the patients ate only cooked meat products although the cooking temperature levels and time could not be specified. The majority of the patients (73.7%, 28/38) ate cooked meat without understanding the risk of contracting BTB from raw meat.

Respondent’s awareness was only significantly associated with age in such a way that individuals between 29–39 years of age (AOR 0.06, 95% CI 0.01–0.53) and elderly, 50+ years (AOR 0.16, 95% CI 0.03–0.78) were less likely to be aware of BTB compared to the 18–29 year olds (Table 1). The risk of milk and meat consumption habits were not significantly associated to age, sex and educational status of the respondents (Table 2).

**Table 1:** Level of awareness on zoonotic tuberculosis among tuberculous lymphadenitis patients in northwestern Ethiopia

Demography factors	Number of respondents	Number of respondents aware of BTB*	COR (95% CI)**	AOR (95% CI)***
Age (years)				
18–28	17	10 (58.8%)	1.0^+^	1.0
29–39	17	2 (11.8%)	0.10 (0.02–0.54)	0.06 (0.01–0.53)
40–50	13	7 (53.8%)	0.82 (0.19–3.50)	1.20 (0.22–5.57)
≥50	23	5 (21.7%)	0.19 (0.05–0.78)	0.16 (0.03–0.78)
Sex				
Male	52	19 (36.5%)	1.0	1.0
Female	18	5 (27.8%)	0.67 (0.21–2.16)	2.88 (0.50–17.0)
Educational status				
Illiterate	21	4 (19.0%)	1.0	1.0
Read and write only	11	4 (36.4%)	2.43 (0.47–12.5)	4.68 (0.66–33.3)
Primary (1–6)	11	4 (36.4%)	2.43 (0.47–12.5)	2.72 (0.44–17.0)
Secondary and above	27	12 (44.4%)	3.40 (0.90–12.8)	2.04 (0.43–9.65)

* Bovine tuberculosis

** Crude odds ratio

*** Adjusted odds ratio

^+^ Reference value (1.0)

**Table 2:** Habit of boiled milk and cooked meat consumption of tuberculous lymphadenitis patients in northwestern Ethiopia

Demography factors	Number of respondents	Consumed boiled milk only	P value	Consumed cooked meat only	P value
Age (years)					
18–28	17	5 (29.4%)	0.939	11 (64.7%)	0.549
29–39	17	5 (29.4%)		9 (53.0%)	
40–50	13	5 (38.5%)		5 (38.5%)	
≥50	23	8 (34.8%)		13 (56.5%)	
Sex					
Male	52	17 (32.7%)	0.960	29 (55.8%)	0.672
Female	18	6 (33.3%)		9 (50.0%)	
Educational status					
Illiterate	21	7 (33.3%)	0.303	10 (47.6%)	0.673
Read and write only	11	4 (36.4%)		6 (54.5%)	
Primary (1–6)	11	1 (9.09%)		5 (45.5%)	
Secondary and above	27	11 (40.7%)		17 (63%)	

P < 0.05 referred as significant

### Tuberculosis in farmers

All the 70 FNA specimens were cultured in LJ media and mycobacterial growth were detected in 57.1% (40/70). The binary and octal description, SITs and lineage or sub-lineage are summarized for each isolate in Tables 3 and 4. Among the 40 isolates, spoligotyping identified a total of 31 different patterns, of which the majority (80.6%, 25/31) were *M. tuberculosis,* and the remaining were M*. africanum* (13.0%, 4/31) and *M. bovis* (6.40%, 2/31). The *M. tuberculosis* strains belonged to Euro-American lineage (68%, 17/25), East-African Indian (24%, 6/25) and Indo-Oceanic (8%, 2/25). Seventeen strains corresponded to the existing patterns in the SITVIT2 database. Fourteen patterns were not in the database are documented as orphans. The most common strains were SIT53 and SIT289, each with 4 isolates.

**Table 3:** Spoligopatterns of shared types and corresponding lineages identified from tuberculous lymphadenitis patients in northwestern Ethiopia

SIT	No. of isolates	SITVIT clad by KBBN	Major lineage by CBN	Octal number	Binary format
37	1	T3	EA	777737777760771	
42	1	LAM	EA	777777607760771	
50	1	H3	EA	777777777720771	
52	1	T2	EA	777777777760731	
53	4	T	EA	503777740003771	
131	1	T3	EA	777717777760771	
134	2	H3	EA	777777777720631	
149	2	T3-ETH	EA	777000377760771	
168	1	H3	EA	777777777720671	
334	1	T	EA	577777777760771	
602	1	H1	EA	777777770000771	
1745	1	T3	EA	773737777760771	
25	2	CAS1-Delhi	EAI	503757740003471	
26	1	CAS1-Delhi	EAI	703777740003771	
289	4	CAS1-Delhi	EAI	703777740003571	
982	1	BOV	*M. bovis*	416773777777600	
665	1	BOV_1	*M. bovis*	616773777777600	

The 40 isolates were grouped into 31 different spoligotype patterns (strains). Of which 17 strains (patterns) have registered in the SITVIT2 database and the remaining 14 patters were orphans and presented in this table. Five patterns were in clusters, containing 14 isolates (2–4 isolates per cluster), and the dominant strains were SIT53 and SIT289 with 4 isolates each. The fifteen strains were belongs to Euro-American (12 patterns) and East-African Indian (3 patterns), and the remaining two strains were belonging to *M. bovis*

*KBBN* knowledge-based Bayesian networks, *CBN* conformal Bayesian network

**Table 4:** Spoligopatterns of orphan strains and corresponding lineages identified from tuberculous lymphadenitis patients in northwestern Ethiopia

SIT	No. of isolates	SITVIT clad by KBBN	Major lineage by CBN	Octal number	Binary format
Orphan	1	Manu2	EA	577747777742670	
Orphan	1	H3-Ural-1	EA	757777777420771	
Orphan	1	T1-RUS2	EA	000000077760771	
Orphan	1	T3-ETH	EA	777000077760771	
Orphan	1	LAM	EA	777777607760771	
Orphan	1	CAS1-Delhi	EAI	503757740003471	
Orphan	1	CAS1-Delhi	EAI	503767744003571	
Orphan	1	CAS1-Kili	EAI	541347400003460	
Orphan	1	AFRI	MA	400000047177671	
Orphan	1	AFRI	MA	700000007177371	
Orphan	1	AFRI	MA	400000047177571	
Orphan	1	Manu_ancestor	MA	715203477377571	
Orphan	1	EAI4-VNM	IO	743777774003571	
Orphan	1	Manu2	IO	757777777423771	

Fourteen of the total 31 strains were identified as orphan strains in the present study and indicated in this table. The orphan strains belonged to four major lineages including Euro-American (5 strains), East-African Indian (3 strains), *M. africanum* (4 strains), and Indo-Oceanic (2 strains)

*KBBN* knowledge-based Bayesian networks, *CBN* conformal Bayesian network

### Tuberculosis in cattle

All the study cattle were tested for BTB with CIDT and interpreted at a ≥4 mm cutoff point. None of the animals were positive for BTB. Further analyses such as microbial cultures and genotypic tests were not conducted to isolate and characterize mycobacteria from cattle tissues. As a result, comparisons with MTBC strains identified in human subjects were not performed.

### Transmission of mycobacteria between farmers and their cattle

The results of this study did not provide evidence of direct transmission of the MTBC species between farmers and their cattle in the smallholder farms of northwestern Ethiopia. However, there is preliminary evidence of zoonotic transmission of *M. bovis* between animals and two human TBLN patients. Further investigations are required to determine whether *M. bovis* is directly transmitted from animals to their owners.

## Discussion

### Level of awareness of the farmers and their food consumption habits

This study evaluates the awareness of respondents as it pertains to BTB and the risk of zoonotic transmission from raw milk and meat consumption habits in relation to their age, sex and educational status. Respondents’ awareness on BTB was generally poor (34.3%, 24/70), and magnifies the public health implication of the disease in the study area. Our finding is consistent with 35, 25.7, 6.9, 29.7 and 15% awareness levels reported in earlier Ethiopian studies [12, 13, 17, 27, 28]. The low level of awareness observed in the present study could also be related to the low level of BTB in the study area. Earlier epidemiological studies have also indicated that the level of disease awareness among famers is related to the prevalence of the disease in that specific area [29] in such a way that awareness on BTB was lower in low prevalent settings compared to high BTB prevalent areas [30].

Awareness of BTB among human TBLN patients in this study was associated with age. Clusters of ages between 29–39 and 50+ years were less likely to be aware of BTB compared to the younger ages (18–29 years). The observed difference in the level of awareness between the different age categories was difficult to interpret. However, it could be related to the expanding nature of education to village level in Ethiopia. As a result, the younger ages have rather more access to education, and had knowledge on BTB and its zoonotic importance compared to the elder one.

The majority (67.1%, 47/70) of the respondents in this study consumed raw milk, and is consistent with the previous study conducted in Central Ethiopia [28], reported 79.3% (46/58) of livestock holders had habit of raw milk consumption. We, therefore, suggest that improving knowledge and awareness of milk borne transmission is important to prevent zoonotic TB in human in the study area.

### Tuberculosis in farmers

Molecular characterization by spoligotyping identified strains of *M. tuberculosis* as the major causative agents of TBLN in human patients participated in this study. This finding is compatible with previous studies in Ethiopia [31–33]. However, *M. bovis* was isolated from the two TBLN cases even though cattle owned by these patients were none reactor for CIDT test. Thus, we suggest the potential zoonotic transmission of *M. bovis* from animal since the two TBLN cases with *M. bovis* were identified by the questionnaire as consumers of raw milk and undercooked meat product.

SIT53 and SIT149 were the dominant strains identified in TBLN patients participated in the present study. These strains also isolated in human FNA and sputum samples collected and studied previously in Ethiopia [32, 34–36], and suggest the presence of similarity in the population of mycobacterial strains that causes PTB and EPTB.

### Tuberculosis in cattle

We did not demonstrate that TB is transmitted from human TBLN patients to their cattle in this study. All cattle were found negative for BTB after CIDT test, and none of the cattle tissue had cultured for mycobacterial growth. However, SIT53 and SIT149, which were isolated from human TBLN patients in the present study, were also isolated from cattle [21, 23] and goat [22] previously in Ethiopia, which indicate possible human-to-animal transmission.

### Transmission of mycobacteria between farmers and their cattle

A potential transmission of zoonotic TB was observed in this study since there was isolation of *M. bovis* from two human TBLN cases even though direct link of transmission between specific cattle and its owners were not confirmed. Similar findings were reported from previous Ethiopian studies [9, 19, 20]. These studies isolated *M. bovis* from human pulmonary TB (PTB, it is an infectious disease principally caused by *M. tuberculosis*, which is primarily a human pathogen and characterized by the growth of tubercles in the tissues, especially the lungs) and/or TBLN cases, and suggested the occurrence of transmission of zoonotic TB between livestock and humans. However, despite close contact between humans and livestock, and low level of awareness of zoonotic TB, including the high consumption rate of raw milk by farmers in the study area, the prevalence of *M. bovis* in human TBLN patients recorded in the present study was lower than expected. This could be related to the overall low prevalence rate of BTB in the study area as it was shown in the present study and 1.27% prevalence reported previously in northwestern Ethiopia [18]. The small sample size (70 TBLN) could also contribute to the recorded lower isolation rate of *M. bovis* from farmers in the study area.

## Conclusions

Our data revealed that many of the study participants were unaware of BTB and its public health consequence. *M. bovis* isolates were identified from two human subjects raising cattle. These subjects were diagnosed for TBLN and there was no evidence that the TBLN was caused by simultaneous presence of *M. tuberculosis*. Although a direct transmission route was not proven here, it is possible that TB caused by *M. bovis* is a zoonotic disease in northwestern Ethiopian. However, further investigations are required to prove a direct transmission route from cattle to owner. Continued education of the public, particularly farmers with livestock, is important to protect the zoonotic pathogen as a potential public health threat in the area.

## Abbreviations

AFB: acid fast bacilli
A-PPD: avian purified protein derivative
B-PPD: bovine purified protein derivative
BCS: body condition score
BTB: bovine tuberculosis
CBN: conformal Bayesion network
CI: confidence interval
CIDT: comparative intradermal tuberculin
DR: direct repeat
EPTB: extra pulmonary tuberculosis
FNA: fine needle aspiration
FNAC: fine needle aspiration cytology
LJ: Lowenstain Jenson
LN: lymph node
M.: mycobacterium
Ml: milliliter
MTBC: mycobacterium tuberculosis complex
PCR: polymerase chain reaction
PPD: purified protein derivative
SIT: shared international type
TB: tuberculosis
TBLN: tuberculous lymphadenitis
